# Prevalence of *Neofusicoccum parvum* Associated with Fruit Rot of Mango in South Italy and Its Biological Control Under Postharvest Conditions

**DOI:** 10.3390/jof11050384

**Published:** 2025-05-17

**Authors:** Laura Vecchio, Alessandro Vitale, Dalia Aiello, Chiara Di Pietro, Lucia Parafati, Giancarlo Polizzi

**Affiliations:** Department of Agriculture, Food and Environment (Di3A), University of Catania, 95123 Catania, Italy; laura.vecchio@phd.unict.it (L.V.); dalia.aiello@unict.it (D.A.); chiara.dipietro@phd.unict.it (C.D.P.); lucia.parafati@unict.it (L.P.) gpolizzi@unict.it (G.P.)

**Keywords:** *Neofusicoccum parvum*, Botryosphaeriaceae, mango fruit decay, biocontrol agents, killer yeasts

## Abstract

Botryosphaeriaceae species were recently found to be responsible for heavy mango crop losses worldwide. In 2020, mango fruit samples showing fruit decay symptoms were collected from Glenn, Kent, Irwin, Palmer, Brokaw 2, and Gomera 3 accessions in 4 orchards located in Sicily (Italy). A molecular analysis of the ITS and tub2 regions performed on 41 representative isolates allowed for the identification of mainly *Neofusicoccum parvum* and occasionally *Botryosphaeria dothidea* (1/41) as the causal agents of fruit decay. Pathogenicity proofs were satisfied for both fungal pathogens. Ripe and unripe Gomera 3 mango fruits were used to compare the virulence among the *N. parvum* isolates. Postharvest experiments performed on Gomera 3 fruits and by using different biocontrol agents (BCAs) showed that the performance of treatments in reducing fruit decay depends on *N. parvum* virulence. The data show that unregistered *Wickerhamomyces anomalus* WA-2 and *Pichia kluyveri* PK-3, followed by the trade bioformulate Serenade™ *(Bacillus amyloliquefaciens* QST713), were the most effective in managing mango fruit rot. This paper shows, for the first time, the potential of different BCAs, including *Trichoderma* spp., for the controlling of postharvest decay caused by *N. parvum* on mango fruits.

## 1. Introduction

Tropical and subtropical crops, like mango (*Mangifera indica* L.), avocado (*Persea americana* Mill.), dragon fruit (*Hylocereus undatus* (Haw.) Britton & Rose), passion fruit (*Passiflora edulis* Sims) and litchi (*Litchi chinensis* Sonn.), are becoming more popular and are rapidly expanding in southern Italy (Sicily; Calabria; Apulia; and, lately, also Sardinia). In Sicily (southern Italy), mango crops play a leading role both for export and local consumption, finding the optimal conditions along the northern coast of the island between the provinces of Messina and Palermo until an altitude of 50–80 m above sea level. The quality and marketability of mango fruits are highly correlated with different factors, such as the maturity stage [1,2], harvesting method [3], postharvest treatment, packaging, handling procedures, and mode of transport [4]. Moreover, the susceptibility of mango fruits to some fungal diseases increases after harvesting and prolonged storage as a result of physiological changes in the fruit promoting pathogen development [5].

The major fungal diseases of mango are caused by *Colletotrichum* spp. [6,7,8,9] and Botryosphaeriaceae species [10,11]. Botryosphaeriaceae species are responsible for aerial symptoms, such as woody cankers, shoot blight, and dieback, as is also the case for other fruit tree hosts [12], and, above all, stem-end rot or fruit rot worldwide. Fruit symptoms can be observed on immature fruit still attached to a plant or during storage at a packing house and in transit [13,14]. Many species have been reported in mango fruits in China, Malaysia, Brazil, India and Australia such as *L. theobromae*, *L. crassispora*, *L. egyptiacae*, *L. hormozganensis*, *L. iraniensis*, *L. pseudotheobromae*, *L. brasiliense* and *L. mahajangana*, *N. parvum* and *N. ribis*, *Fusicoccum aesculin*, *Pseudofusicoccum violaceum*, *P. adansoniae*, *P. ardesiacum*, *P. kimberleyense*, *Neoscytalidium dimidiatum* and *N. novaehollandiae*, *Botryosphaeria scharifii*, *B. dothidea* and *B. ramosa*, *Dothiorella dominicana*, and *D. mangiferae* [11,15,16,17,18,19].

In Italy, fungal species belonging to Botryosphaeriaceae represent the main threat for mango production. Numerous surveys conducted between 2010 and 2021 showed a decline and dieback in young plants and the blight of seedlings in nurseries caused by Botryosphaeriaceae [20,21]. However, mango fruit symptoms caused by this family have not been reported so far in Italy. Thus, a deep investigation is needed to ascertain the spread of Botryosphaeriaceae infections in fruit and to identify the main associated fungal species.

Many studies have reported some chemical methods to manage Botryosphaeriaceae diseases in fruit [22,23,24,25,26,27]. However, the lack of authorized chemical compounds on mango in Italy as well as the requirement to reduce the applications of synthetic compounds according to the European legislation has led us to evaluate eco-friendly alternative control strategies in organic agriculture. Biological control of fruit rot using microbial antagonists is one of the most promising alternatives. However, so far, a few studies have only reported the effects of antagonistic microorganisms, such as *Bacillus* spp. and yeasts [28,29,30], to control stem-end rots and decays caused by *N. parvum*.

Considering the increased spread of mango fruit rot caused by Botryosphaeriaceae species observed in Sicily in the last two years and the limited data available about their biological control, the aims of this study were as follows: (i) to detect, within the Botryosphaeriaceae family, which species are mainly involved as causal agents of fruit rot in different mango fruits in the main representative Italian area of production (eastern Sicily, province of Catania) using morphological characteristics and TUB sequence data; (ii) to preliminarily evaluate the virulence of a representative subset of *N. parvum* isolates in mango fruit; (iii) to assess the performance of few biological products and potential antagonistic wild yeasts in controlling mango fruit rot caused by both the most and the least virulent isolates of *N. parvum* under postharvest conditions.

## 2. Materials and Methods

### 2.1. Field Sampling and Isolation

During 2020, many field surveys were conducted in four mango orchards located in eastern Sicily (province of Catania, Italy). Different mango fruits showing fruit rot were sampled from at least 10 plants of ‘Glenn’, ‘Kent’, ‘Irwin’, ‘Palmer’, ‘Brokaw 2’, and ‘Gomera 3’ accessions and were transferred to the laboratory of Plant Pathology at the Department of Agriculture, Food and Environment of the University of Catania for pathogen isolation. 

To this aim, about 1000 small pieces (about 5 × 5 mm), taken from the margin of symptomatic fruit, were superficially sterilized for 60 s with 1.2% sodium hypochlorite (NaClO), rinsed in sterile deionized water (SDW), dried, and plated on potato dextrose agar (PDA, Lickson, Vicari, Italia) amended with 100 mg L^−1^ of streptomycin sulphate (Sigma-Aldrich, St. Louis, MO, USA). Subsequently, the plates were incubated in the dark at 25 ± 1 °C for seven days. Subsequently, mycelial plugs from the actively growing margins of young colonies were transferred onto new PDA dishes to create pure cultures. To obtain monoconidial cultures, single-hyphal or terminal tips were selected from resulting colonies and transferred into new PDA plates. A total of 220 fungal isolates were collected from different mango accessions. Among these, 41 isolates representative of each accession and orchard were selected on the basis of morpho-biometric features and were stored in the collection of the above-mentioned department before performing further analyses.

### 2.2. DNA Extraction and PCR

The genomic DNA of the representative subset of 41 isolates was extracted using the Wizard Genomic DNA Purification Kit (Promega Corporation, Madison, WI, USA) after they were grown on PDA for seven days. Primers Bt2a and Bt2b [31] and ITS5 and ITS4 [32] were used to amplify up the partial beta tubulin locus (*tub2*) and internal transcriber spacer region (ITS). According to the manufacturer’s instructions, each amplification was performed in a final volume of 25 μL using One Taq™ 2× Master Mix with Standard Buffer (BioLabs, Ipswich, MA, USA) guidelines for using an AG 22371 Eppendorf Mastercycler (Fisher Scientific, Hampton, NH, USA). The PCR cycle was as follows: 30 s at 94 °C for the first 30 cycles, 30 s at 94 °C for 35 cycles, 52 °C (tub2) for 1 min, 1 min at 68 °C, and 5 min at 68 °C. The PCR result was seen on 1% agarose gels at 90 V for 40 min, followed by purification, GelRed™ staining, and sequencing by Macrogen Inc. (Seoul, Republic of Korea). Sequences were visualized and edited using the software MEGA 11: Molecular Evolutionary Genetics Analysis across computing platforms [33]. A BLASTn search of TUB locus was conducted in NCBI to determine the closest relatives.

### 2.3. Pathogenicity Tests

Pathogenicity proofs were carried out with one representative isolate of *N. parvum* and one of *B. dothidea* on three ripe and three unripe mango fruits. Healthy fruits were wounded at two points with a sterile micro-needle, and a mycelial plug with a diameter of 6 mm from a 10-day-old culture of each isolate was placed on each wound of the fruits. The control consisted of wounded and inoculated fruits with PDA plugs. Successively, inoculated mango fruits were placed in a growth chamber at 25 °C and 80% relative humidity. Disease incidence (DI) and symptom severity (SS) were evaluated 3 days after inoculation (dai). The DI value was referred to as the assessment of the percentage of positive inoculation points, whereas the SS was counted at each inoculation point, measuring the mean lesion length based on two perpendicular diameters.

### 2.4. An Assessment of the Virulence of Neofusicoccum parvum

To assess the virulence of *N. parvum*, forty isolates were inoculated, each on three unripe and three ripe mango fruits (i.e., 3 replicates) of cv Gomera 3, respectively. Comprehensively, two-hundred and forty fruits were employed for this experiment. The experiment was performed under the same conditions of the previous one and was repeated once.

### 2.5. Performance Evaluation of Biological Treatments Against Neofusicoccum parvum

Experiment I. A preliminary test to evaluate the effectiveness of biological treatments in reducing fruit rot caused by the most virulent MO1 and the least virulent MF36 isolate was performed. A total of nine products were tested on mango fruit cv Gomera 3. The tested products were six trade bioformulates, two antagonistic yeasts and a chemical standard. The yeast strains used herein belong to the Di3A (Department of Agriculture, Food and Environment, University of Catania, Italy) collection, and they were previously identified as *Wickerhamomyces anomalus* WA-2 and *Pichia kluyveri* PK-3 by sequencing the D1/D2 region of the 26S rRNA gene [34]. Moreover, the selected strains have been shown to exhibit the killer phenotype and have a wide spectrum of both cellular and extracellular enzyme activities [35]. Each yeast strain was routinely maintained at 4 °C on Petri dishes containing Yeast Extract Peptone Dextrose Agar (YPDA; yeast extract, 10 g; peptone, 20 g; dextrose, 20 g; agar, 20 g (Oxoid, Basingstoke, UK) per liter of distilled H_2_O) and was refreshed on the same culture medium prior to use. The scheme of this preliminary experiment was conceived to compare biological treatments with an effective chemical standard (Table 1). The treatments were performed 24 h before or at the same time as the pathogen inoculation. Five mango fruits per treatment were used for each timing, with two points of inoculation for each fruit. The surface of the fruits was disinfected with 70% ethanol, rinsed in SDW, and air-dried. After that, all mango fruits were sprayed with 100 mL of the product solution with the dosages indicated in Table 1. The same procedure was followed to evaluate the antagonistic activity of *W. anomalus* WA-2 and *P. kluyveri* PK-3. Each yeast suspension was prepared in SDW by collecting cells grown in YPD for 48 h at 25 °C and by adjusting the concentration to 10^7^ cells/mL. Successively, each fruit was inoculated with two plugs (6 mm in diameter) of the mycelium of each isolate and was placed in plastic containers and was incubated in a growth chamber with a 12 h photoperiod at 25 ± 1 °C. The mean lesion length (two perpendicular axes or diameters) was measured after 3 days of incubation, and the area of the relative ellipse was calculated according to the following formula: A = (α/2) × (β/2) × π, where A is the area of ellipse, α and β are the two perpendicular axes of ellipse, and π = 3.1415… After incubation, the amount of disease reduction was also calculated and referred to the Abbott Index, AI = [(T − C)/T)] × 100, where T represents the value detected in the untreated fruits, and C represents the value detected in the treated fruits.

Experiment II. Based on previous data, an additional experiment was designed that included only biological treatments, with the exception of the trade citrus essential oil (Prev-Am Plus). Their application was performed twice, 3 days before the inoculation and at the same time of the pathogen inoculation using the same isolates of *N. parvum* used in experiment 1 (MO1 and MF36). This experiment was performed twice and was conducted as described above for the previous one.

### 2.6. Statistical Analysis

Data regarding the preliminary virulence evaluation of 40 isolates of *N. parvum* and the performance of biological treatments on mango fruit cv Gomera 3 were analyzed by using the Statistica Package Software (Vers. 10; Statsoft Inc., Tulsa, OK, USA). The arithmetic means of lesions were calculated by averaging the values for all replicates of each isolate or biological treatment. Initial analyses of the disease lesions on the fruits were performed by calculating associated F and *p* values to evaluate whether the effects of single factors and interactions are significant. In the post hoc analyses, the mean values of the DI and SS parameters were subsequently separated by Fisher’s least significant difference test (*p* = 0.05). For all experiments performed for biological performance evaluation. The percentage of disease reduction was also accounted for [36].

## 3. Results

### 3.1. Pathogen Isolation and Identification

**Figure 1:**
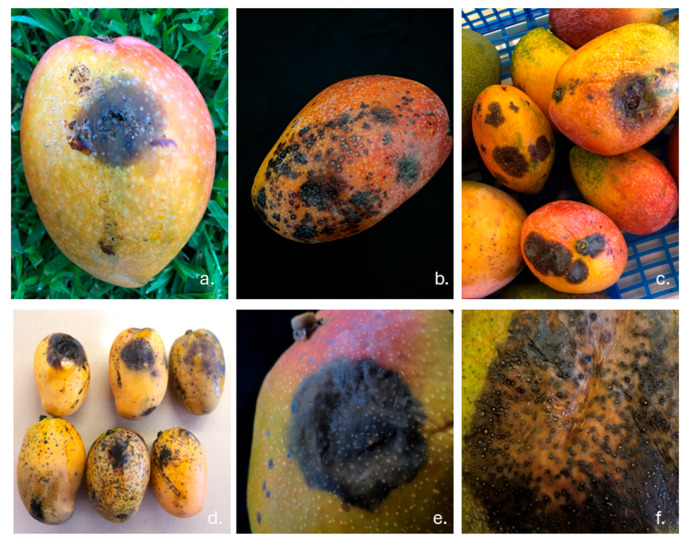
Fruit rot caused by *Neofusicoccum parvum* on mango fruit accessions; (**a**) cv Glenn; (**b**,**c**) cv Irwin; (**d**) cv Gomera 3; (**e**) cv Kent; (**f**) pycnidia on fruits.

All symptomatic mango accessions showed rot symptoms on the stem-end of the fruits or on most of the fruit pericarps (Figure 1). Isolations consistently yielded colonies resembling botryosphaeriaceous fungi, and, in particular, fungal colonies showed a fluffy aerial white–grey mycelium, turning black over time. Based on a morphological characterization coupled with ITS and a *tub2* sequence analysis (Table 2), 40 isolates were identified as *Neofusicoccum parvum* (Pennycook & Samuels) Crous, Slippers, & AJL Phillips and one isolate as *Botryosphaeria dothidea* (Moug.: Fr.) Cesati & De Notaris.

### 3.2. Pathogenicity Tests

Both of the inoculated fungal species (MF17 and MF1 isolates) produced necrotic lesions similar to those observed in the field of each inoculated point of ripe and unripe mango fruits after 3 days. The isolate of *N. parvum* (MF1) produced an average lesion of 1.20 cm for the ripe fruits and 1.03 cm for the unripe fruits, while the isolate of *B. dothidea* (MF17) was able to cause an average lesion of 0.40 cm and 0.27 cm, respectively. The pathogens were re-isolated from symptomatic tissues and identified as described above.

### 3.3. An Assessment of the Virulence of Neofusicoccum parvum

As regards the preliminary assay on the virulence of *N. parvum*, there was a significant effect for isolate and fruit harvesting single factors and for isolate × fruit harvesting interactions on lesion size (Table 3).

Consequentially, the two datasets were analyzed for each fruit harvest (Table 4). A great variability in virulence versus mango fruit was detected among selected *N. parvum* isolates. Isolate MO1 was, on average, the most virulent, whereas MF36 was the least virulent isolate (Table 4). Based on these data, MO1 and MF36 were selected for the following evaluation of the bioformulates’ performance.

### 3.4. Evaluation of Biological Treatments Against Neofusicoccum parvum

Experiment I. A preliminary evaluation of treatment performance in reducing infection caused by both MO1 and MF36 isolates revealed that the effects of treatment and isolate were significant for disease lesion size, whereas the effect of timing on disease infection, i.e., 24 h before inoculation or simultaneously, was not significant (Table 5).

Following the ANOVA test, post hoc comparisons revealed the best performance for fludioxonil (Geoxe™) that was able to significantly reduce nearly to zero disease severity decay (0.9–1.8 mm^2^), whereas the effectiveness of all biological treatments was always lower and very variable in all conditions, i.e., in a preventative or simultaneous application, and by inoculating two *N. parvum* isolates having different virulence values.

Experiment II. Data analysis regarding the evaluation of the performance of biological products in reducing decay caused by MO1 and MF36 isolates showed that the effects of single factors were significant for disease lesion size and also for treatment × isolate interactions (Table 6). For this reason, data were analyzed separately for each isolate.

The ranking of performance among the biological control agents and significant differences among them are separately reported for *N. parvum* isolates in Table 7.

**Figure 2:**
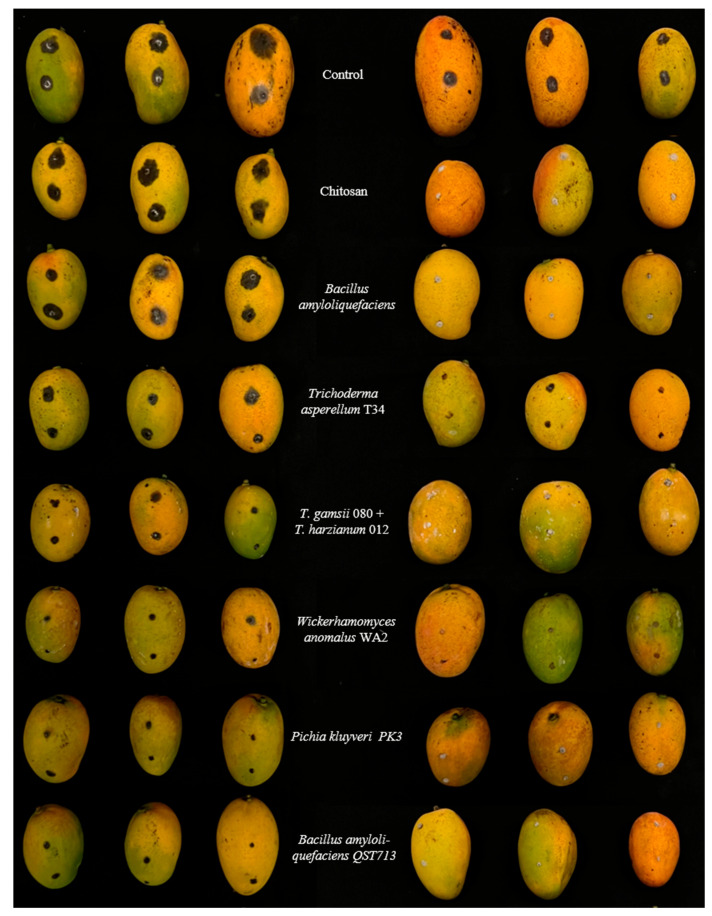
Postharvest effects of biological products and wild yeasts on mango fruit cv Gomera 3 against the most virulent *Neofusicoccum parvum* isolate MO1 (on the **left**) and the less virulent isolate MF36 (on the **right**).

Based on these data (Table 7), all biological treatments were able to significantly reduce the fruit decay (AI of more than 50%) caused by the least virulent *N. parvum* isolate MF36. However, a few differences were detected among the treatments. In detail, *T. asperellum* T34 (T34 Biocontrol) was the least effective, whereas *B. amyloliquefaciens* QST713 (Serenade), *W. anomalus*, and *P. kluyveri* PK3 were the most effective in reducing fruit decay. Under high infection levels (isolate MO1), chitosan (Prevatect), *B. amyloliquefaciens* (Amylo-X), *T. asperellum* T34 (T34 Biocontrol), and the *T. gamsii* + *T. harzianum* trade mixture (Remedier) failed to significantly reduce the average fruit lesion size. Only *B. amyloliquefaciens* QST713 (Serenade), *W. anomalus*, and *P. kluyveri* PK3 gave the best reductions (AI values of around 70%), thus reconfirming the performance results obtained with MF36. An exemplificative overview of these data is also reported in Figure 2, in which both the behavior between isolates at different virulence values and performance variability among the biological treatments are clearly visible.

## 4. Discussion

In this paper, we first identified the diversity within the Botryosphaeriaceae family that is responsible for fruit decay, i.e., among a subset of *N. parvum* isolates, obtained from different mango fruits in one of the most representative Italian production areas (eastern Sicily, province of Catania) by using morphological characteristics and ITS and tub2 sequence data. Although it is well known that besides *N. parvum*, other different genera and species (*Lasiodiplodia theobromae*, *B. dothidea*, *Diaporthe* spp.) are involved in infections of the above-ground parts of mango [20,21], our findings clearly demonstrate that *N. parvum* is the most prevalent species involved in mango fruit decay. Indeed, surveys on several accessions clearly show that *N. parvum* is the species that is more frequently associated with fruit rot of mango in Italy, whereas *B. dothidea* has rarely been recovered. This is not surprising, since other surveys have already confirmed that *N. parvum* is widely spread in the Mediterranean basin, and it is able to infect other host species [37,38,39,40,41], including avocado [42].

Moreover, our findings show differences in virulence within an Italian subset of 40 *N. parvum* isolates from mango fruits, and this behavior variability strictly depended on the tested isolate and the maturity stage of the fruits. In detail, the data show a higher susceptibility of ripe fruits. Similarly, Baskarathevan et al. [43] and Puig et al. [44] detected virulence variability among isolates of *N. parvum* tested in pathogenicity tests on green shoots of grapevines and cacao fruit, respectively.

Determining the main species responsible for fruit infections is the first step to developing efficacy control strategies. Since fungicide application is limited in the EU by restrictive regulations, and potential biological products to be used in pre- and postharvest conditions are not yet available on the market, the application of biological products or antagonistic microorganisms could represent an attractive alternative for the sustainable management of diseases on mango commodities.

Overall, this work reported on the performance of antagonistic microorganisms and chitosan in reducing the amount of postharvest fruit rot. Serenade™ and *W. anomalus* WA-2 and *P. kluyveri* PK-3 were revealed to be the most effective against the fruit SER of mango, reducing the amount of decay in all experiments, whereas T34 Biocontrol, Prevatect™, Remedier™, and Amylo-X™ were the least effective. This clearly shows that pathogen virulence is a crucial factor that can affect the performance of biological products, which in turns can also depend on antagonist microorganism-targeted phytopathogenic fungus interactions. The potential of yeasts as biological control agents has been reported previously by Carvalho Castro et al. [45], who evaluated the efficacy of yeast in pre- and postharvest conditions on cultivar Tommy Atkins fruits, which were treated with yeasts different from the ones tested here before the inoculation with *N. parvum* and *L. theobromae*. In a more recent paper, *Saccharomyces* spp. and *P. kudriavzeviii* showed good activity in controlling postharvest fruit rot of mango [30]. Otherwise, the performance of *W. anomalus* WA-2 and *P. kluyveri* PK-3 against *N. parvum* has not been previously reported. Both *W. anomalus* and *Pichia kluyveri* are generally recognized as safe (GRAS) microorganisms and are not associated with human pathogenicity. Their safety profile and potential probiotic properties [34], combined with their metabolic versatility, supports their application in food-related environments, including postharvest protection and fermentation processes [46,47,48].

The antagonistic activity of the selected yeasts is likely attributed to their killer phenotype [34], as has been reported for other microorganisms [6,49,50]. In fact, several studies have shown that yeast strains with extracellular glucanase activity can inhibit a wide range of postharvest pathogens in different fruits and through various modes of application [35,51]. Moreover, besides the production of glucanase enzymes, several putative direct biocontrol mechanisms, such as the production of volatile compounds, the competition for space and nutrients, and wound colonization, could also be involved [52]. Regarding the latter, the strong colonization ability of *W. anomalus* may also contribute significantly to its biocontrol efficacy. As reported by Taïbi et al. [53], *W. anomalus* was able to dominate the fruit microbiota, replacing other yeast species after 14 days of mango storage. This capacity to establish and persist on the fruit surface reinforces its role as a competitive and resilient antagonist. In our study, this characteristic likely contributed to the observed biocontrol performance. Furthermore, *W. anomalus* has also been shown to activate plant defense responses [51,54], thus demonstrating its ability to modulate key defense-related enzymes, such as peroxidase, which plays a central role in the induction of systemic resistance. As regards antagonistic bacteria, *Pseudomonas chlororaphis* PCL1606 and *Bacillus velezensis* UMAF6639 have provided encouraging results for disease control in mango and avocado fruits [55].

Based on our data, the timing of treatment applications is another crucial aspect for improving the performance of these products. When applied 3 days before pathogen inoculation, the biological products and yeasts were more effective, while when applying one day before or at the same time as the pathogen inoculation, they can be ineffective. Some authors have previously reported on the in vivo and in vitro efficacy of four species of *Trichoderma* spp. against stem-end rot caused by *L. theobromae* and *N. parvum* on avocado fruit [56]. However, to our knowledge, these findings are the first data on the efficacy of *Trichoderma*-based trade formulates against *N. parvum* causing fruit rot of mango. Further studies should be performed for a large-scale evaluation of the effectiveness of these biological products against natural *N. parvum* infections in field conditions and following postharvest storage conditions.

## 5. Conclusions

We demonstrated herein that biological control in postharvest is potentially achievable for the pre- and postharvest fungal decay of mango fruit. However, the choice of biological product should be modulated according to the infection pressure exerted by the *N. parvum* isolate. In summary, the trade *B. amyloliquefaciens* QST713-based formulate and unregistered *W. anomalus* WA2 and *P. kluyveri* PK3 yeasts could be potentially suggested under any phytosanitary conditions, whereas other *B. amyloliquefaciens*- and *Trichoderma*- and chitosan-based formulates should alternatively be used under postharvest conditions involving low disease pressure. Although further studies should be performed to confirm these findings, this paper contributes to the application of environmentally friendly biofungicides as alternatives to chemicals in ever-increasing tropical commodities worldwide, such as mango fruit.

## Figures and Tables

**Table 1:** Trade and no trade bioformulates and wild yeasts used for in vivo experiments.

Active Ingredient	Trade Name	Manufacturer	Rate (g or L/hL)
Chitosan	Prevatect	Ascenza Italia S.r.l. (Saronno, Italy)	4 L/hL
*Bacillus amilyloquefaciens* QST 713	Serenade ASO	Bayer CropScience S.r.l., Milano—Italy	0.8 L/hL
*Bacillus amyloliquefaciens*	Amylo-X	Biogard, Brussels—Belgium	250 g/hL
*Trichoderma gamsii* 080 + *T. harzianum* 012	Remedier	Gowan Italia S.r.l. (Faenza, Italy)	250 g/hL
*Trichoderma asperellum* T34	T34 Biocontrol	Cifo S.r.l (San Giorgio di Piano, Italy)	25 g/hL
Citrus essential oil	Prev-am Plus	Ascenza Italia S.r.l. (Saronno, Italy)	0.8 L/hL
Fludioxonil	Geoxe (50% a.i)	Syngenta Italia S.p.A., Milano, Italy	100 g/hL
*Wickeramomyces anomalus* WA-2	Unregistered	Department Agriculture, Food and Environment, Università di Catania (Catania, Italy)	10^7^ cells/mL
*Pichia kluyveri* PK-3	Unregistered	Department Agriculture, Food and Environment, Università di Catania (Catania, Italy)	10^7^ cells/mL

**Table 2:** Fungal isolates collected in this study and their GenBank accession numbers.

				GenBank Acc. Numb.	
Species	Strain	Host	Origin	ITS	*tub2*
*Neofusicoccum parvum*	MF1	*Mangifera indica*	Sicily (Italy)	PQ098068	PQ066804
*N. parvum*	MF2	*M. indica*	Sicily (Italy)	PQ098069	PQ066805
*N. parvum*	MF3	*M. indica*	Sicily (Italy)	PQ098070	PQ066806
*N. parvum*	MF5	*M. indica*	Sicily (Italy)	PQ098071	PQ066807
*N. parvum*	MF6	*M. indica*	Sicily (Italy)	PQ098072	PQ066808
*N. parvum*	MF7	*M. indica*	Sicily (Italy)	PQ098073	PQ066809
*N. parvum*	MF8	*M. indica*	Sicily (Italy)	PQ098074	PQ066810
*N. parvum*	MF10	*M. indica*	Sicily (Italy)	PQ098075	PQ066811
*N. parvum*	MF11	*M. indica*	Sicily (Italy)	PQ098076	PQ066812
*N. parvum*	MF13	*M. indica*	Sicily (Italy)	PQ098077	PQ066813
*N. parvum*	MF14	*M. indica*	Sicily (Italy)	PQ098078	PQ066814
*N. parvum*	MF16	*M. indica*	Sicily (Italy)	PQ098079	PQ066815
*Botryosphaeria dothidea*	MF17	*M. indica*	Sicily (Italy)	PQ098067	PQ066844
*N. parvum*	MF18	*M. indica*	Sicily (Italy)	PQ098080	PQ066816
*N. parvum*	MF22	*M. indica*	Sicily (Italy)	PQ098081	PQ066817
*N. parvum*	MF25	*M. indica*	Sicily (Italy)	PQ098082	PQ066818
*N. parvum*	MF26	*M. indica*	Sicily (Italy)	PQ098083	PQ066819
*N. parvum*	MF27	*M. indica*	Sicily (Italy)	PQ098084	PQ066820
*N. parvum*	MF28	*M. indica*	Sicily (Italy)	PQ098085	PQ066821
*N. parvum*	MF29	*M. indica*	Sicily (Italy)	PQ098086	PQ066822
*N. parvum*	MF31	*M. indica*	Sicily (Italy)	PQ098087	PQ066823
*N. parvum*	MF32	*M. indica*	Sicily (Italy)	PQ098088	PQ066824
*N. parvum*	MF33	*M. indica*	Sicily (Italy)	PQ098089	PQ066825
*N. parvum*	MF35	*M. indica*	Sicily (Italy)	PQ098090	PQ066826
*N. parvum*	MF36	*M. indica*	Sicily (Italy)	PQ098091	PQ066827
*N. parvum*	MF39	*M. indica*	Sicily (Italy)	PQ098092	PQ066828
*N. parvum*	MF41	*M. indica*	Sicily (Italy)	PQ098093	PQ066829
*N. parvum*	MF42	*M. indica*	Sicily (Italy)	PQ098094	PQ066830
*N. parvum*	MF44	*M. indica*	Sicily (Italy)	PQ098095	PQ066831
*N. parvum*	MF45	*M. indica*	Sicily (Italy)	PQ098096	PQ066832
*N. parvum*	MF46	*M. indica*	Sicily (Italy)	PQ098097	PQ066833
*N. parvum*	MO1	*M. indica*	Sicily (Italy)	PQ098098	PQ066834
*N. parvum*	MO2	*M. indica*	Sicily (Italy)	PQ098099	PQ066835
*N. parvum*	MO5	*M. indica*	Sicily (Italy)	PQ098100	PQ066836
*N. parvum*	MO7	*M. indica*	Sicily (Italy)	PQ098101	PQ066837
*N. parvum*	MO9	*M. indica*	Sicily (Italy)	PQ098102	PQ066838
*N. parvum*	MO11	*M. indica*	Sicily (Italy)	PQ098103	PQ066839
*N. parvum*	MO12	*M. indica*	Sicily (Italy)	PQ098104	PQ066840
*N. parvum*	MO14	*M. indica*	Sicily (Italy)	PQ098105	PQ066841
*N. parvum*	MO15	*M. indica*	Sicily (Italy)	PQ098106	PQ066842
*N. parvum*	MO18	*M. indica*	Sicily (Italy)	PQ098107	PQ066843

**Table 3:** Analysis of variance for virulence based on average lesion length caused by 40 different Neofusicoccum parvum isolates on mango cv Gomera 3 through 2 fruit harvests.

Factor(s)	Lesion Length (cm) ^1^		
	df	*F*	*p* Value
Isolate	39	25.152	*<0.0001*
Fruit harvesting	1	155.278	*<0.0001*
Isolate × fruit harvest	39	9.971	*<0.0001*

^1^ *F* test of fixed effects; df = degrees of freedom; *p* value associated with *F*.

**Table 4:** Post hoc analyses of differences in virulence of 40 isolates of *Neofusicoccum parvum* inoculated on immature (green) and mature fruits of mango cv Gomera 3.

Isolate	Average Lesion Length (cm) ^1^	
	Ripe	Unripe (Green)
MF1	1.2 ± 0.1 e–j	1.0 ± 0.03 e–g
MF2	0.2 ± 0.03 rs	0.5 ± 0.1 k–n
MF3	1.1 ± 0.1 f–l	0.9 ± 0.2 f–i
MF5	0.5 ± 0.1 p–s	0.6 ± 0.1 i–l
MF6	0.9 ± 0.1 j–o	0.5 ± 0.1 k–o
MF7	1.3 ± 0.1 d–h	1.1 ± 0.03 ef
MF8	0.6 ± 0.1 n–q	1.5 ± 0.1 c
MF10	1.4 ± 0.1 c–g	0.9 ± 0.1 f–i
MF11	0.4 ± 0.1 q–s	0.2 ± 0.1 o–q
MF13	1.3 ± 0.3 d–h	0.9 ± 0.1 f–i
MF14	1.6 ± 0.1 b–d	1.2 ± 0.1 de
MF16	1.7 ± 0.2 bc	1.8 ± 0.2 ab
MF18	0.9 ± 0.03 i–n	0.3 ± 0.03 m–q
MF22	1.4 ± 0.1 c–f	1.0 ± 0.3 e–g
MF25	1.4 ± 0.2 c–g	0.5 ± 0.1 k–n
MF26	1.1 ± 0.1 g–l	1.4 ± 0.03 cd
MF27	0.5 ± 0.03 o–r	0.4 ± 0.03 l–p
MF28	1.0 ± 0.03 h–m	0.3 ± 0.03 m–q
MF29	0.5 ± 0.03 o–r	0.7 ± 0.1 h–k
MF31	1.1 ± 0.2 f–k	0.6 ± 0.1 i–l
MF32	1.2 ± 0.1 e–i	0.4 ± 0.1 l–p
MF33	0.7 ± 0.1 m–q	0.8 ± 0.03 g–j
MF35	1.3 ± 0.1 d–h	0.3 ± 0.03 n–q
MF36	0.2 ± 0.03 s	0.1 ± 0.0 q
MF39	0.4 ± 0.1 q–s	0.1 ± 0.03 q
MF41	0.8 ± 0.03 l–q	0.5 ± 0.1 k–n
MF42	1.2 ± 0.03 e–i	0.6 ± 0.03 j–m
MF44	1.3 ± 0.03 d–h	1.5 ± 0.3 bc
MF45	0.7 ± 0.1 m–q	0.7 ± 0.1 h–l
MF46	1.3 ± 0.1 d–h	0.6 ± 0.03 j–m
MO1	1.6 ± 0.1 b–d	2.0 ± 0.1 a
MO2	0.8 ± 0.1 k–p	0.3 ± 0.03 m–q
MO5	1.4 ± 0.1 c–g	0.3 ± 0.1 m–q
MO7	1.6 ± 0.3 b–d	0.2 ± 0.03 pq
MO9	1.0 ± 0.1 h–m	1.4 ± 0.03 cd
MO11	1.5 ± 0.03 c–e	1.6 ± 0.03 bc
MO12	2.1 ± 0.1 a	1.0 ± 0.03 e–h
MO14	1.1 ± 0.1 g–l	0.5 ± 0.03 k–n
MO15	1.9 ± 0.3 ab	0.9 ± 0.1 f–i
MO18	1.1 ± 0.1 f–l	0.7 ± 0.03 h–k

^1^ Combined data from two experiments. For each experiment, data were derived from three fruits. Standard error of the mean = SEM. Means, followed by different letters within the column, are significantly different according to Fisher’s least significant differences test (α = 0.05).

**Table 5:** ANOVA effects of single factors on disease infections caused by two Neofusicoccum parvum isolates at different virulence values (MO1 and MF36) on mango fruit cv Gomera 3.

Factor(s)	Lesion Area (mm^2^) ^1^		
	df	*F*	*p* Value
Treatment	9	7.7534	*<0.0001*
Isolate	1	68.6299	*<0.0001*
Timing	1	0.0873	0.767996 ^ns^

^1^ *F* test of fixed effects; df = degrees of freedom; *p* value associated with *F*; ns = not significant data.

**Table 6:** Analysis of variance for lesion size caused by *Neofusicoccum parvum* MO1 (most virulent) and MF36 (least virulent) on mango fruit cv Gomera 3 through different biological treatments.

Factor(s)	Lesion Area (mm^2^) ^1^		
	df	*F*	*p* Value
Treatment	7	7.036	*<0.0001*
Isolate	1	90.7813	*<0.0001*
Treatment × isolate	7	2.3026	*0.03*

^1^ *F* test of fixed effects; df = degrees of freedom; *p* value associated with *F*.

**Table 7:** Post hoc analyses of performance of biological treatments in reducing infections in mango fruit cv Gomera 3 caused by MO1 and MF36 isolates at different virulence values.

Isolate	MO1 (Most Virulent) ^1^		MF36 (Least Virulent) ^1^	
Treatment	Lesion (mm^2^)	Reduction (%)	Lesion (mm^2^)	Reduction (%)
Untreated–uninoculated	151.6 ± 13.5 a	-	53.8 ± 12.3 a	-
Chitosan (Prevatect™)	110.4 ± 34.3 a	27.2	15.1 ± 2.1 bc	71.9
*B. amyloliquefaciens* (Amylo-X™)	107.7 ± 20.2 a	28.9	12.3 ± 2.8 bc	77.2
*T. asperellum* T34 (T34 Biocontrol™)	105.3 ± 14.2 a	30.5	26.4 ± 3.4 b	50.9
*T. gamsii* + *T. harzianum* (Remedier™)	104.8 ± 24.8 a	30.9	14.4 ± 2.8 bc	73.2
*Wickerhamomyces anomalus* WA-2	36.8 ± 6.9 b	75.7	6.1 ± 2.7 c	88.7
*Pichia kluyveri* PK-3	39.8 ± 9.2 b	73.7	10.5 ± 0.9 c	80.5
*B. amyloliquefaciens* QST713 (Serenade™)	46.5 ± 10.1 b	69.3	9.4 ± 1.9 c	82.5

^1^ Combined data from two experiments. For each experiment, data were derived from five replicates each formed by two mango fruits. Standard error of the mean (±SEM). Means followed by different letters within the column are significantly different, according to Fisher’s least significant differences test (α = 0.05).

## Data Availability

Data are available on request to the corresponding author.
